# Decentralized Distributed Sequential Neural Networks Inference on Low-Power Microcontrollers in Wireless Sensor Networks: A Predictive Maintenance Case Study

**DOI:** 10.3390/s25154595

**Published:** 2025-07-24

**Authors:** Yernazar Bolat, Iain Murray, Yifei Ren, Nasim Ferdosian

**Affiliations:** 1 School of Electrical Engineering, Computing and Mathematical Sciences, Curtin University, Perth, WA 6102, Australia; nasim.ferdosian@curtin.edu.au; 2 School of Minerals, Energy, and Chemical Engineering, Curtin University, Perth, WA 6102, Australia; i.murray@curtin.edu.au; 3 VeilNet Pty Ltd., Perth, WA 6164, Australia; yifei.ren@veilnet.org

**Keywords:** edge AI, distributed inference, neural network partitioning, tiny machine learning, IoT, deep neural network, wireless sensor networks, predictive maintenance

## Abstract

The growing adoption of IoT applications has led to increased use of low-power microcontroller units (MCUs) for energy-efficient, local data processing. However, deploying deep neural networks (DNNs) on these constrained devices is challenging due to limitations in memory, computational power, and energy. Traditional methods like cloud-based inference and model compression often incur bandwidth, privacy, and accuracy trade-offs. This paper introduces a novel Decentralized Distributed Sequential Neural Network (DDSNN) designed for low-power MCUs in Tiny Machine Learning (TinyML) applications. Unlike the existing methods that rely on centralized cluster-based approaches, DDSNN partitions a pre-trained LeNet across multiple MCUs, enabling fully decentralized inference in wireless sensor networks (WSNs). We validate DDSNN in a real-world predictive maintenance scenario, where vibration data from an industrial pump is analyzed in real-time. The experimental results demonstrate that DDSNN achieves 99.01% accuracy, explicitly maintaining the accuracy of the non-distributed baseline model and reducing inference latency by approximately 50%, highlighting its significant enhancement over traditional, non-distributed approaches, demonstrating its practical feasibility under realistic operating conditions.

## 1. Introduction

DNNs are advancing towards increasingly complex model structures with deeper layers and large model sizes, making them highly effective for many applications such as healthcare, smart homes, autonomous driving, smart cities, and many more. One key application is anomaly detection in industrial settings, where DNNs excel at identifying unusual patterns that may indicate potential equipment failures [1]. Microcontroller unit (MCU)-based IoT devices are gaining popularity across various industries due to their low power consumption and efficient operation [2]. These IoT devices generate vast amounts of data, which require sophisticated processing to extract meaningful insights. Deep Learning (DL), a subset of Artificial Intelligence (AI), is commonly employed to handle this data volume, as it can learn patterns and uncover valuable insights from complex datasets [3].

Nevertheless, deploying a large deep neural network (DNN) on IoT edge devices, particularly tiny microcontrollers with limited energy, processing power, and memory, presents a considerable challenge due to the high computational demands and significant memory and power requirements of such models [4]. One approach to this problem is to perform the part of DNN inference on the edge while offloading the remaining computations to the cloud, achieved by strategically partitioning the model along an edge-cloud continuum [5,6,7,8]. However, sending every second of sensed data to the cloud consumes network bandwidth, computing resources, and drains battery power, while also raising privacy concerns due to the potential exposure of sensitive data. Additionally, processing huge amounts of data in the cloud poses a difficulty, especially for industry applications such as underground mines and maritime, where communication is not always feasible [9].

Recent advancements in AI have further enabled the execution of machine learning models on resource-constrained, tiny devices, leading to the emergence of Tiny Machine Learning (TinyML) [10]. To enable TinyML at the edge, compression techniques such as quantization [11], pruning [12], and knowledge distillation [13] can be used to reduce model size and optimize performance on limited hardware. These methods can shrink the model to a manageable size, enabling efficient execution on edge devices. However, when high compression rates are necessary, the accuracy of the compressed DNN model may be significantly compromised [14,15].

A potential approach to enable DNN at the edge is distributing the computational load of DNN inference across multiple edge devices. This method allows large DNN model deployment without the need for model compression, which preserves accuracy. Furthermore, it enhances responsiveness and privacy by eliminating the need for a cloud server during model inference [16]. However, none of the existing distributed inference methods have explored the potential of distributing neural network inference specifically to achieve high accuracy, particularly for resource-limited devices deployed within wireless sensor networks (WSNs). Most existing distributed approaches primarily aim to maximize inference throughput by leveraging relatively powerful edge devices, such as Raspberry Pi or NVIDIA Jetson [17,18,19,20]. These approaches typically involve centralized coordination, complex partitioning, and parallel processing, and require frequent and continuous synchronization among nodes. Consequently, they introduce substantial computational and communication overhead, making them unsuitable for deployment on tiny devices with limited computational capabilities.

To address these challenging issues, we proposed a novel approach, named Decentralized Distributed Sequential Neural Network (DDSNN), explicitly designed for deployment on resource-constrained microcontroller devices within WSNs. DDSNN is an end-to-end neural network distribution method to enable DNN inference on resource-limited tiny devices equipped with only a few kilobytes of RAM. Our distribution method preserves full 32-bit floating-point precision without employing aggressive model compression techniques, which are commonly used in TinyML applications. To accomplish this, we adopt sequential layer-wise partitioning where the pre-trained DNN is split at natural layer boundaries, and the resulting sub-models are deployed across multiple MCUs. These sub-models collectively replicate the inference capability of the original neural network by operating sequentially, enabling direct inter-device communication within the WSN without requiring a central coordinator or dispatcher. This strategy significantly reduces computational overhead and communication demands, making it particularly suitable for deployment in low-power, resource-limited WSN environments. In summary, this research paper provides the following contributions:

- Our primary contribution is introducing a fully decentralized, distributed inference solution named DDSNN specifically suited for practical deployment on resource-constrained, low-power MCUs in actual wireless sensor network environments.
- The proposed approach achieves high inference accuracy under strict TinyML constraints without depending on intensive model compression.
- The practical feasibility and robust accuracy of the proposed DDSNN are empirically validated in a real-world predictive maintenance scenario, utilizing low-power MCUs and actual wireless communication protocols for equipment monitoring through vibration analysis. The DNN model, trained on real-world vibration data, is partitioned and independently implemented on each MCU, allowing devices to collaboratively monitor equipment conditions and issue real-time alerts.
- Experimental results confirm that our DDSNN achieves a remarkable accuracy of 99.0%, highlighting its feasibility and reliability in realistic operating conditions.

The rest of the paper is organized as follows: Section 2 provides some background on neural network-based anomaly detection and reviews the existing approaches from cloud-centric solutions to TinyML and distributed inference. It highlights the limitations of existing methods and identifies key research gaps in deploying advanced neural networks on highly resource-constrained edge devices. Section 3 details the design of the proposed DDSNN approach. Section 4 describes the implementation, and Section 5 presents evaluation methods, while Section 6 presents experimental results and analysis. Finally, Section 7 provides the conclusion and outlines future research directions.

## 2. Related Work

Early neural network-based anomaly detection approaches leveraged cloud technologies combined with edge and fog computing paradigms [21,22]. Cloud-assisted approaches often rely on unpredictable cloud availability and suffer from latency, and offloading data to the cloud increases privacy concerns [23].

With the evolution of TinyML, various quantization and pruning techniques have been proposed to enable ML model execution on tiny microcontroller devices [24]. As a result, several studies have managed to deploy lightweight neural networks by reducing the complexity of the model through feature reduction. Some of the works that implemented TinyML for anomaly detection in industry are [25,26,27,28]. Yet, the trade-off between accuracy loss and model size should be well balanced when compressing the model for a smaller footprint. Although TinyML enables AI at the edge for tiny devices, it suffers from resource constraint issues, which prevent the deployment of complex DNNs that offer high accuracy, automatic feature extraction, and many more [29].

Current studies on DNN distributed inference use two main types of distribution: layer-pipelined (sequential) distribution and hybrid (layer-pipelined + intra-layer parallel) distribution. Layer-pipelined (sequential) distribution refers to methods that partition a neural network strictly at the layer boundaries, assigning distinct groups of layers to individual devices arranged sequentially. Each device completes its assigned part before passing the intermediate output to the next device. Once the pipeline is fully utilized, each device processes different input samples simultaneously, enabling all nodes to be active concurrently. However, each neural network layer resides entirely within a single device, meaning no layer is split across multiple devices. By contrast, hybrid distribution involves an additional partitioning strategy known as intra-layer splitting, also called horizontal partitioning or model-parallel splitting. In intra-layer splitting, computationally intensive layers, such as convolutional layers, are partitioned into multiple segments (e.g., by splitting feature maps or channels) and processed simultaneously across several devices. Thus, hybrid distribution combines sequential processing across layers with parallel processing within individual layers [16].

Most of the studies in the current literature have focused on the hybrid execution of inference [17,20,30,31,32,33,34,35]. Hybrid or parallel distribution can improve throughput and reduce overall latency for high-computation tasks by distributing the load. However, it often requires more frequent synchronization and communication between nodes, especially in scenarios where outputs from different parts must eventually be merged. Given its high communication and computation demands, this distribution method is better suited for more powerful devices with reliable, high-speed network connections, making it less suitable for resource-constrained devices in WSNs. Consequently, studies exploring parallel distribution primarily use more powerful embedded devices, such as mobile phones, as shown in Table 1. Authors in [36] attempted to reduce inter-device communication by taking advantage of Knowledge Distillation, where the original teacher Neural Network is partitioned based on its knowledge, and small student networks learn only a specific part of the teacher’s function. Furthermore, [37] extends this work, making Network of Neural Networks (NoNN) more robust to communication failures by improving its partitioning technique and suggesting a new partitioning approach. In both cases, although this method reduces inter-device communication, the compression of the model by training child nodes can lead to a high dependency on the teacher network’s training dataset.

Unlike parallel distribution, relatively few studies explored layer-pipelined sequential distribution [18,19,38,39]. For instance, studies [18,19] propose a sequential partitioning approach using Directed Acyclic Graphs (DAG), where a central dispatcher partitions the DNN model and assigns segments to individual nodes at runtime orchestration. Each node processes its assigned segment, and final outputs must be returned to the dispatcher for merging, increasing synchronization complexity and communication overhead. The earlier work [18] introduced this dispatcher-based approach but did not systematically optimize partitioning decisions. The extended version [19] improved this by developing a rigorous graph-based optimization algorithm to identify optimal partitioning points and node placement, significantly reducing bottleneck latency and enhancing overall inference throughput. While this refined method offers notable advantages for complex scenarios, particularly in contexts where different edge devices perform smaller, interdependent tasks in parallel, it also introduces substantial operational complexity. Specifically, their runtime dispatcher-based orchestration strategy involves continuous synchronization among nodes, dynamic partitioning adjustments to accommodate changing computational loads or network conditions, and centralized coordination to manage data flow and task allocation. Furthermore, the continuous and intensive communication necessitated by centralized orchestration substantially increases communication overhead, demanding frequent exchanges of control and intermediate data among nodes. These demands exceed the limited capabilities and energy constraints of resource-constrained, battery-operated microcontrollers operating in WSNs. Similarly, authors in [39] adopt a cluster-based computing strategy relying on relatively powerful edge devices. This method employs MPI-based communication typically over wired Ethernet networks, making it less applicable to tiny microcontrollers due to its computational and communication demands.

**Table 1:** Related work summary in distributed inference.

Ref.	Paper (Year)	Dist Method	Intra-Layer Split	Communication	Hardware	Decentralized	Infer on Edge	Tiny ML
[39]	AutoDiCE (2023)	Layer-pipeline		MPI over Ethernet	Jetson Xavier/TX2	✓	✓	✗
[20]	DeeperThings (2021)	Hybrid	fused-tile split	Ethernet	Raspberry Pi 3–Pi 4	✗	✓	✗
[18]	DEFER (2022)	Layer-pipeline	-	Simulated (CORE emulator)	Simulation of Jetson nodes	✗	✗	✗
[30]	DISNET (2023)	Hybrid	micro-split chosen at runtime	Wi-Fi	RPi 4 + Jetson Nano	✓	✓	✗
[17]	DistrEdge (2022)	Hybrid	DRL may split layers	Wi-Fi	Jetson Nano + RPi	✓	✓	✗
[38]	DN^2^PCIoT (2019)	Layer-pipeline	-	Simulation	MCUs (sub-MB SRAM)	✓	✗	✓
[40]	PANCODE (2023)	Layer-pipeline	-	Simulation	Same MCU class as above	✓	✗	✓
[33]	Optimised YOLO (2021)	Hybrid	conv split between Pi and Intel NCS	Wired Network	Raspberry Pi 4 + NCS-2	✗	✓	✗
[19]	Joint Partition & Placement (2022)	Layer-pipeline	-	Wired Network	Simulation only	✓	✗	✗
[34]	Collaborative Inferencing (2020)	Hybrid	spatial/channel/filter splits	Wi-Fi	Raspberry Pi 3B	✓	✓	✗
[41]	Automated Partitioning (2024)	Layer-pipeline	-	Wired Network	Simulation on ASIC	✓	✗	✗
[35]	HiDP (2025)	Hybrid	split an input or feature map	Wi-Fi	NVIDIA Jetson + RPi	✗	✓	✗
	DDSNN (our work)	Layer-pipeline	-	Wi-Fi	ESP32-S3 MCU	✓	✓	✓

Among these works, only [38] explicitly targets resource-constrained devices to optimize distributed pipeline inference throughput. They employ a sophisticated, fine-grained, graph-based partitioning algorithm, where the neural network is modeled as a dataflow graph with vertices corresponding to individual neurons or layers. Their optimization method explicitly searches for optimal CNN partitioning by systematically swapping vertices, carefully considering resource constraints such as memory usage and communication overhead. The authors further extended their approach by employing a multilevel strategy to reduce computational complexity and enabling both vertical and horizontal partitioning through their hybrid Operator-Attribute-Parameter (OAP) representation [40]. However, despite its theoretical strength and general applicability, this approach remains largely validated through simulations without empirical deployment on actual hardware. Moreover, their approach remains highly customized, relying on complex vertex operations that typically exceed the computational capabilities of most tiny microcontrollers, thereby creating significant deployment constraints. Additionally, their assumptions regarding ideal network conditions limit the practical applicability of their theoretical estimations, reducing their effectiveness in real-world scenarios. In contrast, our method provides an accessible end-to-end solution achievable through widely used, publicly available frameworks such as TensorFlow and Keras, significantly enhancing practical usability and compatibility with resource-constrained microcontrollers.

While these existing approaches share certain conceptual similarities with our work in terms of employing sequential inference pipelines, they commonly rely on cluster-based computing architectures, centralized coordination mechanisms, wired communication infrastructures, or simulation-based evaluations, as can be noticed from Table 1. Such characteristics significantly limit their applicability to WSN environments. Our work differentiates itself from these existing studies by directly addressing specific challenges in WSN environments, such as limited computational power and memory capacity, strict energy constraints demanding efficient local processing, and the absence of centralized control. This capability significantly enhances the practical feasibility and effectiveness of deploying advanced neural network inference in resource-constrained WSN applications. To demonstrate the practical effectiveness of our proposed approach, we validate it through real-world experiments rather than relying solely on simulations. The DNN model, trained on real-world vibration data, is partitioned and independently implemented on each MCU, allowing devices to collaboratively monitor equipment conditions and issue real-time alerts.

Moreover, it is important to highlight that DDSNN is not limited to a specific application domain. Like other distributed inference methods, our DDSNN framework can be broadly applied across various real-world scenarios. For instance, prior distributed inference studies demonstrate diverse applications, such as distributed image classification [17], distributed object detection [33], collaborative inference for smart-camera networks and urban surveillance [34], and decentralized intelligence for fire surveillance using low-power edge devices in smart city infrastructures [42]. These examples illustrate how decentralized deep neural networks, including DDSNN, can deliver robust, scalable, and energy-efficient solutions across a wide range of applications, such as public safety, urban monitoring, and surveillance, thereby reinforcing their generalizability and broad adaptability beyond predictive maintenance alone.

## 3. Methodology

This section details the methodology and system architecture of our proposed DDSNN approach. Specifically, we describe our sequential layer-level partitioning strategy, define the optimization constraints guiding the partitioning process, and outline the overall distributed system architecture.

### 3.1. Partitioning Method

**Figure 1:**
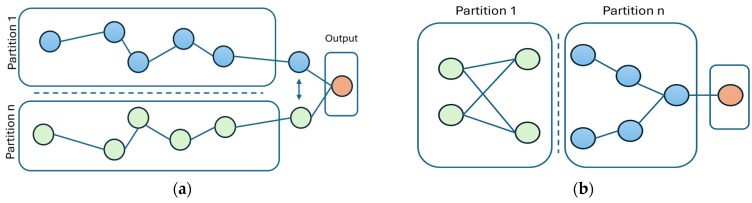
(**a**) Partitioning method: neuron-level partitioning; (**b**) layer-level partitioning.

When partitioning DNN for inference, two primary methods are typically employed: neuron-level and layer-level partitioning [16], as depicted in Figure 1. Neuron-level partitioning (Figure 1a) operates at a fine-grained scale, dividing individual layers by splitting neurons, channels, or spatial dimensions. This fine granularity offers improved load balancing, as it distributes computational tasks more evenly across nodes. However, this approach can significantly increase communication overhead due to frequent synchronization of intermediate outputs between nodes, especially in convolutional layers where data dependencies are high. In contrast, layer-level partitioning (Figure 1b) segments the neural network into distinct groups of complete layers. Each partition processes its assigned layers sequentially and passes the resulting intermediate output to the subsequent partition. While this method reduces communication frequency, thus decreasing overhead, it might lead to uneven computational load distribution across partitions, especially when layer complexities vary significantly. Layer-level partitioning, however, is generally simpler to implement and more suitable for resource-constrained environments due to its lower synchronization requirements.

**Figure 2:**
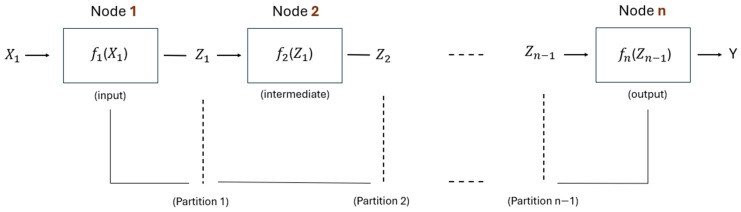
Sequential neural network partitioning using Functional Composition representation for n nodes.

Our work utilizes layer-level partitioning with a sequential distribution approach. By applying layer-wise partitioning, we split the pre-trained global model sequentially at layer boundaries, resulting in sub-models that each encompass a contiguous set of layers from the original neural network. Unlike works such as [38,40], which employ fine-grained DAG-based partitioning, our method adopts a simpler yet highly efficient layer-level sequential partitioning strategy. DAG-based methods represent neural networks as Directed Acyclic Graphs, with each neuron or operation as a graph vertex and data dependencies as edges. This fine-grained representation requires careful dependency tracking and synchronization between nodes, introducing significant overhead due to increased buffering, frequent communication, and runtime complexity, which is especially challenging for resource-constrained IoT devices. We take advantage of the natural layer-by-layer structure of neural networks and represent the neural network as a Functional Composition representation. Each function represents a single layer or a group of multiple layers in the neural network. Partitioning, therefore, consists of splitting the network *F* into sequential sub-functions $f_{1}$, $f_{2}$, and $f_{n}$, deployed, respectively, on Node 1, Node 2, and Node n, as illustrated in Figure 2.

- Node 1: Receives the initial input dataset $X_{1}=\;\left\{x_{1,1},\;x_{1,2},\ldots ,\;x_{1,n},\right\}$ and processes it using the function $f_{1}$, generating intermediate output $Z_{1}=\;\left\{z_{1,1},\;z_{1,2},\ldots ,\;z_{1,n},\right\}=\;f_{1}(X_{1})$. Here, $f_{1}$ contains layers assigned to Node 1, and the resulting output $Z_{1}$ becomes the input to the subsequent function $f_{2}$ in Node 2.
- Node 2: Receives intermediate results $Z_{1}$ from Node 2 and processes them using a function $f_{2}$, encompassing the layers within Node 2. This yields another intermediate output $Z_{2}=\;\left\{z_{2,1},\;z_{2,2},\ldots ,\;z_{2,n},\right\}=\;f_{2}(Z_{1})$. The processed intermediate result $Z_{2}$ from Node 2 is forwarded to the following node.
- Node n: This final node receives the intermediate data $Z_{n-1}$ transmitted by Node n−1. Using function $f_{n}$, which includes the last group of neural network layers, it completes the inference process by generating the model’s final predictions: $Y=\;\left\{y_{1},\;y_{2},\ldots ,\;y_{n}\right\}=\;f_{n}(Z_{n-1})$.

Consequently, the entire distributed neural network F can be explicitly formulated as a sequence of function compositions, partitioned and executed sequentially across the n nodes:

(1) $$F\left(X_{1}\right)=\;f_{n}\;\times \;f_{n-1}\;\times \;\ldots \;\times \;f_{2}\times \;f_{1}\left(X_{1}\right)=\;f_{n}\left(\;f_{n}\left(\ldots {(f}_{2}\left(f_{1}\left(X_{1})\right)\right)\;\ldots \right)\right)$$

### 3.2. Optimization Problem for Partitioning

The partitioning of deep neural networks is driven by the need to distribute the inference load across multiple constrained microcontroller units (MCUs), referred to as nodes. The goal is to ensure that each partition fits within the hardware constraints of the individual node while maintaining the overall model accuracy and minimizing communication overhead. We defined three main constraints that must be addressed in partitioning:

1. Node Capacity Constraint.

Each node has a limited memory capacity, denoted by M. To ensure that a partitioned sub-model fits on a node, the total memory required for both its parameters and all the associated data must not exceed this capacity. For a node N with a set of layers $L_{N}$ assigned to it, we formalize the constraint as follows:

(2) $$\sum_{I\in L_{N}}\left(W_{I}+C_{I}\right)\leq M,$$

where

- $W_{I}$ is the memory required to store weights and biases (i.e., parameters) of layer l;
- $C_{I}$ represents the total memory needed to hold the intermediate data generated by layer l. It accounts for both input buffers (to receive data from preceding layers), and output buffers (to store the activation values or feature maps produced by the layer).

2. Input and Output Dimensions Consistency.

To ensure smooth data flow between nodes and maintain data integrity, we must guarantee that each sub-model’s output shape precisely matches the subsequent sub-model’s input shape. Let $S_{1},\;S_{2\;},\ldots ,\;S_{n}$ represent the sub-models, where n is the total number of sub-models after partitioning. $O_{i}$ represents the output of sub-model i and $I_{i+1}$ represents the input of the sub-model i+1. The function $f_{i}$ represents the computation of the sub-model $S_{i}$. The output of a sub-model must match the input of the next sub-model:

(3) $$O_{i}=f_{i}(I_{i})$$

For consistency between models, we require that the output of the i-th sub-model equals the input of the (i +1)-th sub-model:

(4) $$O_{i}=I_{i+1}$$

This ensures that the dimensions and data passed from one model to the next are compatible, preventing feature loss. Additionally, let the $d(O_{i})$ and $d(I_{i+1})$ denote the dimensions of $O_{i}$ and $I_{i+1}$, respectively. To avoid dimensional mismatches, we require:

(5) $$d\left(O_{i}\right)=d(I_{i+1})$$

If $S_{1}$ outputs a feature vector with shape (32, 128), then the input of $S_{2}$ must also accept a feature vector of shape (32, 128). This consistency holds across all sub-models:

(6) $$d\left(O_{1}\right)=d\left(I_{2}\right),\;d\left(O_{2}\right)=d(I_{3}),\ldots ,d\left(O_{n-1}\right)=d(I_{n})$$

Ensuring feature integrity throughout the partitioned system. The final output $O_{n}$ of the last sub-model $S_{n}$ should be the same as the output of the non-partitioned model:

(7) $$O_{n}=\hat{y}$$

where $\hat{y}$ is the prediction of the original model, ensuring that the partitioned model produces the same result as the non-partitioned original model.

3. Communication Overhead.

One key challenge in partitioning is minimizing the communication overhead between devices, as this can introduce latency and bottlenecks. The communication cost can be formalized as the amount of data transferred between MCUs at each partition point. Let $D_{i}$ denote the size of the data output from the sub-model i that must be transferred to the sub-model i+1. The total communication complexity $T_{comm}$, is given by the following:

(8) $$T_{comm}=\sum_{i=1}^{k-1}D_{i}$$

The goal is to minimize $T_{comm}$ by keeping intermediate outputs $D_{i}$ as small as possible without sacrificing feature integrity. This can be accomplished by strategically choosing partition points at layers that significantly reduce data dimensionality or complexity. Therefore, the optimization problem is formulated as follows:

(9) $$\mathrm{Minimize}\;T_{comm}\;\mathrm{subject}\;\mathrm{to}\;W+C<M\;\mathrm{and}\;O_{n}=\hat{y}$$

### 3.3. Proposed System Architecture

**Figure 3:**
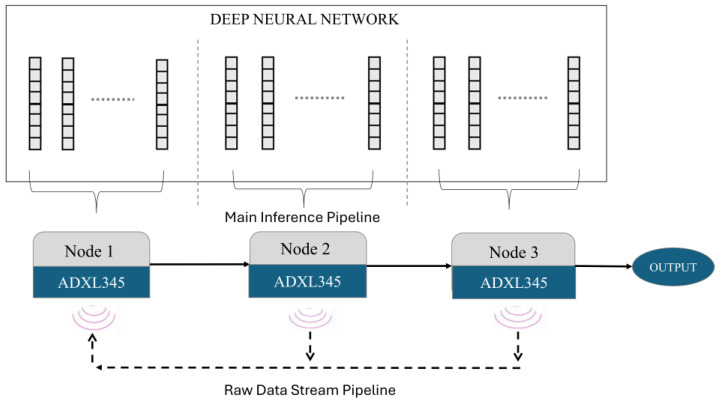
Overall system architecture.

The overall architecture of our anomaly detection system for predictive maintenance, based on the DDSNN, is depicted in Figure 3. As defined earlier in Section 3.1, the pre-trained DNN is partitioned across three nodes in a sequential pipeline—each node runs a different group of layers and passes its output to the next node. This layer-by-layer pipeline means each device only communicates with its immediate neighbor, eliminating the need for centralized coordination. This configuration avoids complex communication paths or backtracking, significantly reducing communication overhead and computational load on the microcontrollers, aligning well with our objective of running DNN inference efficiently within TinyML constraints.

Additionally, for our predictive maintenance scenario, we equip both intermediate (Node 2) and final (Node 3) nodes with ADXL345 accelerometer sensors. Each node independently measures vibrations, allowing the system to detect anomalies from multiple locations on a machine. We refer to this scenario as a Distributed Multi-Source Anomaly Detection setup. This approach is particularly valuable for large machines, where edge nodes can be strategically placed across different parts of the machine to provide comprehensive monitoring. By detecting localized vibrations that may not impact the overall machine condition, the system offers a more detailed and nuanced assessment of the machine’s health. In this setup, we have established two distinct pipelines: our main pipeline, which processes data using partitioned sub-models, and an additional raw data stream pipeline designed for transmitting raw data from downstream nodes back to the head node (Node 1). The head node always receives input data, and the final node generates the anomaly predictions as output. Each node is equipped with an ADXL345 accelerometer sensor to sense vibrations continuously. The intermediate and final nodes transmit their local vibration measurements directly to the head node (Node 1), where the combined data streams are sequentially processed through the distributed neural network pipeline. Thus, the raw data collected by the first node and the data forwarded from subsequent nodes flow through the distributed pipeline, and the final node produces the anomaly detection results.

### 3.4. Pipeline Parallelism

Our architecture further enhances inference throughput by ensuring all nodes remain continuously occupied through pipeline parallelism. Instead of allowing nodes to idle while waiting for a single chunk to traverse the entire pipeline, our approach leverages parallel processing by enabling each node to immediately process new data chunks as soon as the previous chunk is forwarded. When employing pipeline parallelism in distributed sequential neural network inference, a critical problem arises due to the continuous transmission of inference chunks without proper flow control or buffer management mechanisms. Specifically, intermediate nodes in the pipeline can become bottlenecks, as they may receive chunks faster than they can process them. This situation can lead to buffer overflow, increased latency, computational overhead, and ultimately reduced inference throughput.

Existing methods addressing this challenge vary significantly. Approaches such as those presented in [30,34] utilize periodic, high-level telemetry to dynamically balance workloads across nodes, proactively redistributing tasks to avoid bottlenecks. However, these solutions rely on relatively powerful, multi-watt Linux-based single-board computers (SBCs), like Raspberry Pi or Jetson devices, capable of handling orchestrator threads and task queues. In contrast, methods like DN2PCIoT [38] and PANCODE [40] implement first-in-first-out (FIFO) queues by relying entirely on TCP’s built-in flow control. In their setup, each node forwards intermediate activations over TCP sockets to subsequent nodes, leveraging TCP’s sliding window and ACK mechanisms. If a downstream node becomes busy or its receive buffer is nearly full, TCP’s sliding window and ACK mechanism automatically slows the sender, preventing buffer overflows and idle waiting. However, these works never instantiate an actual distributed runtime environment, relying instead on analytical pipeline models. In that context, they implicitly assume ideal or constant-bandwidth network conditions and do not discuss runtime back-pressure.

Our experimental results indicate that exclusively relying on TCP’s built-in flow control in practical deployments introduces significant overhead on resource-constrained devices, primarily due to frequent acknowledgment messages, retransmissions, and buffer management delays. Instead, we introduce an application-layer controlled flow management strategy, incorporating deliberate idle delays at the initiating node of the pipeline. This fixed-delay approach significantly reduces the reactive congestion-control triggers inherent in TCP, ensuring smoother pipeline operation and improved inference throughput. Specifically, we use a simple and effective analytical approach to determine the optimal fixed delay between sending consecutive inference chunks from the first node. Given a neural network partitioned sequentially into K nodes, the optimal delay is defined by identifying the node with the maximum service time, as this node sets the throughput limit for the entire pipeline:

(10) $$SEND\text{\_}DELAY=\frac{T_{max}}{W}$$

where the service time $T_{i}$ for each node is defined by the following:

(11) $$T_{i}=\;C_{i}+\;L_{i}$$

Here $C_{i}$ is the experimentally measured computational time at node i, and $L_{i}$ is the transfer time required to send intermediate activations from node i to node i+1. W is the desired pipeline depth, usually equal to the number of nodes in the pipeline. By setting a fixed delay, we space out sending intervals, avoiding rapid bursts of data that could overload the buffers of downstream nodes.

## 4. Implementation

To demonstrate the effectiveness of our DDSNN approach, we implemented it in a predictive maintenance scenario, analyzing vibration data to detect anomalies in a water pump. CNNs and LSTM-based autoencoders are widely recognized as effective for anomaly detection in time-series data [43]. However, deploying an LSTM-based autoencoder onto low-power microcontrollers proved challenging, as TensorFlow Lite does not fully support complex recurrent architectures, especially those involving dynamic tensor shapes and advanced recurrent operations found in LSTMs [44]. Such operations often exceed the limited computational and memory capabilities of resource-constrained devices, causing deployment failures or runtime errors. Consequently, we selected a CNN-based model, specifically employing a customized LeNet architecture. In our experiment, we focus on partitioning the model across three nodes. We could also explore multiple node configurations, similar to [38]’s work, which has experimented with a wide range of node configurations to observe how inference throughput scales when more devices are added or removed. However, our primary objective here is not to maximize inference throughput but to achieve high accuracy on highly resource-constrained microcontrollers through distributed inference. Adjusting the exact number of nodes does not affect the final accuracy, provided each node can accommodate its allocated sub-model layers.

### 4.1. Experimental Setup

Our setup employs ESP32-S3 Sense microcontrollers sourced from SEEED (Shenzhen, China) [45], paired with ADXL345 accelerometers [46]. Each microcontroller provides 512 KB of internal SRAM, a capacity commonly utilized in TinyML applications. Additionally, the ESP32-S3 offers 8 MB of external PSRAM, which enables us to deploy the original non-partitioned model entirely on a single device, serving as a baseline for further evaluations. Moreover, our selected ESP32-S3 device supports TensorFlow Lite for microcontrollers, which is essential for running machine learning models on resource-constrained devices. Its limited memory and computational capacities make it particularly suitable for evaluating DDSNN in the TinyML domain, specifically addressing the primary challenge of overcoming strict resource constraints inherent to TinyML deployments. It is important to note that our DDSNN approach is not inherently limited to any specific hardware platform. It can be deployed on any MCU that supports TensorFlow Lite operations, as our primary deployment method involves converting models into TensorFlow Lite format for edge deployment. This approach provides significant flexibility in hardware selection, leveraging widely available and publicly accessible frameworks like TensorFlow. For inter-device communication, we utilize the ESP32’s Wi-Fi connectivity to implement a decentralized Wireless Mesh Network (WMN), assigning each node a unique IP address to enable direct communication without requiring a central hub. During inference, the node holding the earlier partition sequentially transmits its intermediate outputs to the subsequent node via TCP, facilitating efficient data sharing. This mesh structure enables each node to act as both a transmitter and a receiver, minimizing communication overhead and optimizing data flow. Moreover, it supports our Distributed Multi-Source Anomaly Detection setup by enabling middle and tail nodes to forward their collected data to the head node while simultaneously receiving outputs from preceding nodes, thus creating a flexible and responsive distributed pipeline.

### 4.2. Data Collection

**Figure 4:**
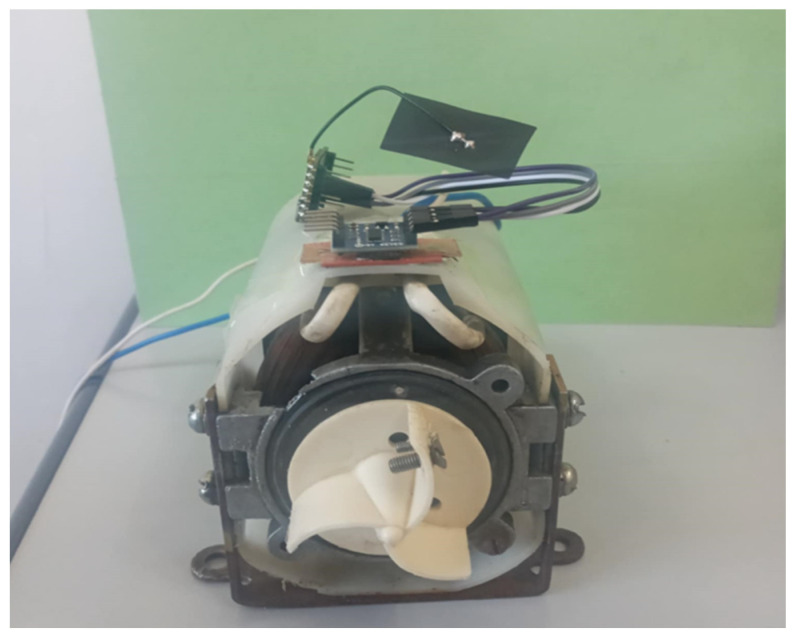
Water pump for data collection.

In our data collection process, an industrial water pump is employed to generate vibration data. To thoroughly evaluate the performance of our distributed neural network, various loads are applied to the motor, producing vibrations at three distinct intensity levels: low, medium, and high. These intensity variations are crucial, as they simulate diverse operational conditions typically encountered in real-world scenarios, enabling precise assessment of our DDSNN’s sensitivity and accuracy. To generate these controlled vibrations, screws of varying sizes are attached to the rotating part of the motor as adjustable loads. The weight of these screws introduces imbalances, shifting the center of rotation and disrupting smooth motor operation, as shown in Figure 4.

### 4.3. LeNet Architecture

**Figure 5:**
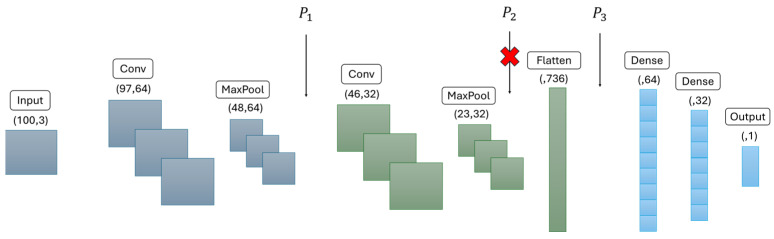
Custom LeNet model and chosen partition points.

Figure 5 illustrates the general architecture of our original LeNet model. Our model features two Conv1D layers for feature extraction and MaxPooling1D layers for dimensionality reduction. The network then flattens the data, passes it through two fully connected Dense layers with Dropout for regularization, and finally outputs a binary classification using a sigmoid activation function.

The Need for Neural Network Partitioning: Our custom LeNet architecture consists of 56,289 trainable parameters, resulting in a total model size of approximately 226.00 KB. Our first deployment attempt on our experimental board with 512 KB of SRAM failed due to insufficient available memory. Specifically, during TensorFlow Lite inference, the system required 49,664 bytes of runtime memory, but only 38,064 bytes were available, leaving an 11,600-byte shortfall. This discrepancy highlights that a significant portion of the MCU’s memory was already consumed by essential system tasks, program code, and runtime overhead, drastically limiting the memory available for the model. Similarly, the SwiftNet Cell architecture, despite having roughly 250 KB of parameters, experienced a peak memory usage of approximately 351 KB, exceeding the limits of an MCU with 512 KB of SRAM [47].

Identifying Partition Points: In line with our optimization problem defined in Section 3.2, our primary objective is to minimize data transfer between devices while respecting each device’s computational capacity and ensuring input-output consistency across partitioned models. This can be achieved by selecting partition points where intermediate dimensions are reduced. As shown in Figure 5, after the pooling and flattening layers, the data dimensions are significantly reduced, resulting in smaller intermediate outputs, thus making these layers ideal candidates for partitioning points ($P_{1}$, $P_{2}$ and $P_{3}$). We decided to omit *P2* because the flattened output at point *P3* is smaller, leading to lower data transfer overhead compared to *P2*. Additionally, convolutional blocks, each consisting of convolutional and pooling layers, demand substantial computational resources, and their workloads are not evenly balanced [48]. Placing both convolutional blocks onto a single device could potentially exceed its resource limits, violating our constraint criteria W+C <M, especially since the first node must also manage incoming data. Consequently, we selected two final partition points: *P1* and *P3*. In further studies of this method, we could develop an algorithm that determines partition points based on our defined optimization constraints. However, since our current LeNet model is relatively straightforward and specifically optimized for low-power devices, developing an algorithm is not necessary at this stage.

### 4.4. Layer-Wise Partitioning in TensorFlow

We implemented Neural Network partitioning directly in Keras and TensorFlow. To partition the pre-trained LeNet model, we use Python slicing (model.layers[:3]) to extract relevant layers from the original model and manually create sub-models. For each sub-model, we are rebuilding it by adding the relevant layers of the original model. Each created sub-model is then compiled separately so that it can be used independently. Compilation is only required so that the sub-models can accept input and generate output. It does not modify the weights of the model. The weights are preserved from the original model when the layers are sliced and added to each sub-model. Therefore, the performance should remain unchanged as long as the original weights are used and not modified. The only reason we need to compile each sub-model is to ensure it can be used for prediction, but we are not re-training the sub-models, which is key to ensuring the performance remains identical to the original model.

### 4.5. DDSNN Deployment and Distributed Inference

**Figure 6:**
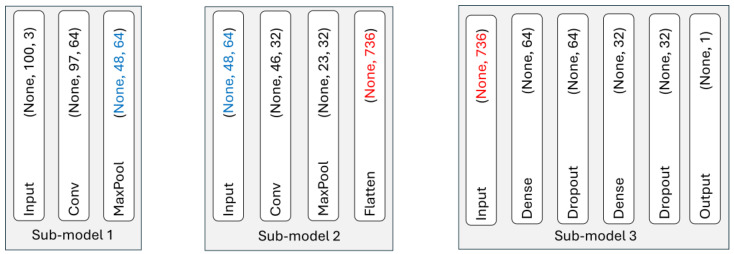
Distributed LeNet Inference Data Flow.

The partitioning process of the original LeNet model generated three independent sub-models. According to our input and output dimension consistency optimization problem defined in Section 3.2, each partition of the LeNet is structured so that its output tensor seamlessly becomes the input to the subsequent node. Since sequential neural networks do not automatically propagate input-output dimensions, we explicitly defined the input layer of each sub-model based on the output shape of the preceding sub-model. As shown in Figure 6, Node 1 processes a (100, 3)-shaped input and produces an output tensor of shape (48, 64). This serialized intermediate result, with embedded shape information, is passed to Node 2, which deserializes the data and continues the network flow through another convolution block and flattening layer, yielding a 736-dimensional vector. Node 3 reconstructs that vector and feeds it into the final dense layers to generate a single scalar prediction (e.g., anomaly vs. normal).

Additionally, for each sub-model, we set the batch size to be dynamic, as it is more memory-efficient for resource-limited devices to process one sample at a time. We then converted each sub-model into TensorFlow Lite format for Microcontrollers using 32-bit floating-point precision, preserving the original model accuracy without applying quantization. The conversion process produced C header files containing model weights in binary form, which were then embedded directly into the deployment scripts of each node. Each sub-model was deployed individually onto its respective node. The deployment scripts were coded to transmit these intermediate outputs via TCP to the corresponding subsequent node in the distributed inference pipeline. By preserving dimensional consistency at each boundary, the distributed architecture mimics the original LeNet end-to-end, ensuring accurate and efficient inference despite the model being split across multiple nodes.

## 5. DDSNN Evaluation

The primary goal of our evaluation is to demonstrate that our distributed partitioning method effectively preserves the accuracy originally obtained during model training when performing inference directly on edge devices. Typically, when deploying trained deep neural networks onto resource-constrained edge hardware, issues such as limited computational resources, memory constraints, or the need for model compression often result in reduced accuracy. Although the accuracy achieved during training may vary depending on the chosen dataset or DNN architecture, our key contribution is maintaining this original accuracy during real-world, on-device inference at the edge using our distributed inference approach.

### 5.1. DDSNN Inference Evaluation

Memory-Efficient Binary Serialization: To serialize our data, we use binary serialization, a format well suited for our constrained devices since it minimizes the computational overhead when transmitting intermediate results. This format not only conserves memory but also enables each data field to be distinctly labeled. We take advantage of this labeling when measuring performance metrics. To calculate the accuracy of our DDSNN predictions, we must identify each predicted sample and compare it to the true label. Therefore, to track the samples as they pass through the distributed inference pipeline, we implement the following strategy:

- Using the binary serialization data field, we assign each processed sample a unique ID and an incremental position index indicating the current node. As a sample moves from Node 1 (index 0) to Node 2 (index 1) and finally Node 3 (index 2), its ID remains unchanged. This step is crucial for matching each final prediction to the correct label and accurately computing the confusion matrix upon completing all samples.
- The test dataset, exported as a header file, is integrated into Node 1’s script, which processes inputs in chunks. Meanwhile, a true labels header file is placed in the final node to match each prediction with its corresponding label, thus objectively evaluating model accuracy.

### 5.2. Test Scenarios

Our LeNet model was originally designed as a binary classifier to differentiate between normal and abnormal conditions without considering different vibration intensities. To increase evaluation complexity, we modified its final layer to a SoftMax activation, enabling multi-class classification among low, medium, and high vibration anomalies. Additionally, we quantized the original LeNet model using a compression method known to have the least impact on accuracy and compared its performance with our DDSNN approach. Specifically, we employed full-integer post-training quantization, converting all weights and biases from 32-bit floating-point to 8-bit integer values, and calibrated the quantized model using a representative dataset. This quantization technique generally results in minimal accuracy degradation compared to other compression methods [49]. We evaluate and compare the performance of the quantized int8 LeNet model and our 32-bit floating-point DDSNN approach under both binary and multi-class classification tasks. We benchmark these two approaches against the non-partitioned original LeNet model deployed on a single device, which serves as our baseline. To deploy the original LeNet as a 32-bit floating-point model, we utilize the external PSRAM of our MCU, as its primary SRAM is insufficient for running the unquantized and non-partitioned model.

### 5.3. Inference Throughput and Latency Measurement

We measure the inference time of our distributed sequential pipeline using a round-trip latency measurement approach. This strategy was adopted due to challenges in precisely synchronizing the timing across multiple nodes within a wireless sensor network, particularly given the absence of any centralized dispatcher or timing controller in our decentralized setup. Node 1 acts as the master node, initiating inference by forwarding input data sequentially through Node 2 to Node 3. Immediately after completing inference, Node 3 sends a brief acknowledgment (ACK) message containing the processed chunk identifier back to Node 1. Upon receiving this ACK, Node 1 calculates the total round-trip latency by recording timestamps at transmission and upon ACK reception. From this measured latency, we explicitly subtract the minor ACK latency, which was independently determined through controlled ping-pong measurements, to accurately isolate the pipeline’s inference latency. Our practical evaluations were conducted in a typical home Wi-Fi network environment (operating at a 40 MHz bandwidth, with approximately 100 Mbps throughput), providing realistic insights into distributed inference performance.

**Figure 7:**
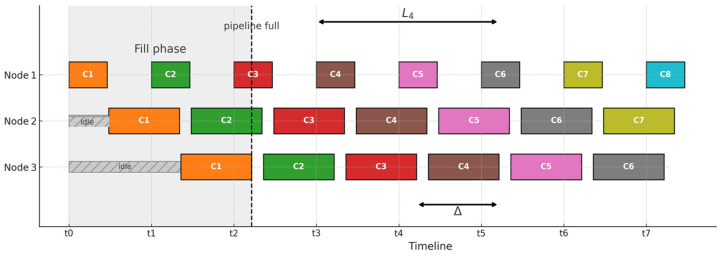
Pipeline timeline illustrating end-to-end latency and inference throughput. Six colored chunks (C1–C6) propagate through the three-node pipeline. The arrow marked $L_{4}$ shows the complete end-to-end latency of chunk C4—from its dispatch on Node 1 to its completion on Node 3. The horizontal arrow labeled Δ spans the interval between the completion of C3 and C4 on Node 3, representing the steady-state gap that governs sustained inference throughput.

We measure two inference times in our distributed pipeline: inference throughput and end-to-end latency. Figure 7 illustrates these two metrics in detail. At the start of the pipeline operation, only Node 1 is active, processing chunk C1, while Nodes 2 and 3 remain idle, awaiting data. As soon as Node 1 completes processing chunk C1 and forwards it to Node 2, it immediately starts processing the next chunk (C2). This sequential forwarding of chunks continues, progressively occupying Nodes 2 and 3.

The pipeline reaches a fully occupied state when all nodes (Node 1, Node 2, Node 3) simultaneously process different chunks, indicated by the vertical dashed line labeled “pipeline full.” Once the pipeline is fully occupied, it transitions into the steady-state phase, enabling parallel processing. In this state, each node consistently processes different inference chunks simultaneously, maximizing resource utilization.

- **End-to-end latency,** represented by the $L_{4}$ arrow in Figure 7, measures the total duration for a single inference chunk (C4 in this case) to propagate through the entire pipeline, from the moment it enters Node 1 until processing completes on Node 3.
- **Pipeline inference throughput** measures how quickly the pipeline continuously delivers inference results once all nodes become fully occupied and operate simultaneously (steady state). At a steady state, inference completions occur at consistent intervals, denoted as Δ. To accurately measure this throughput, Node 1 records timestamps whenever it receives acknowledgment (ACK) messages for completed inferences. The time differences between consecutive ACKs give us the steady-state interval Δ, clearly indicating how long each inference travels through the entire pipeline during ongoing, continuous operation.

Lastly, we measure and compare the inference latency of the quantized int8 LeNet and the original LeNet models against the inference throughput achieved by our DDSNN approach.

### 5.4. Power Consumption

**Figure 8:**
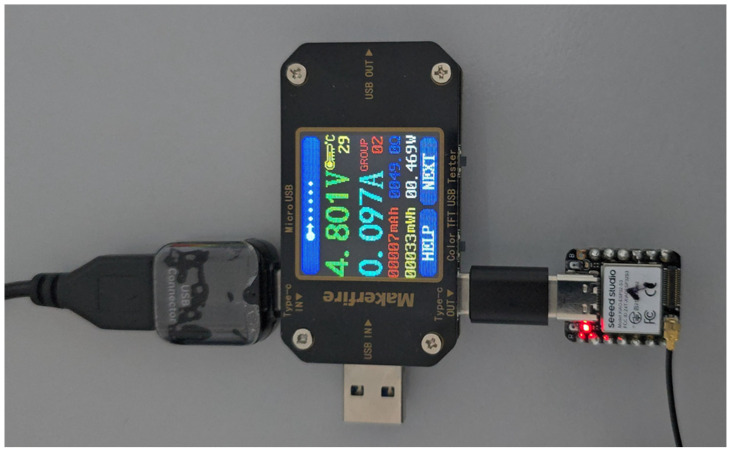
Energy measurement via MakerHawk digital multimeter.

In our experiment, we measure per-node power consumption during on-device inference. To accurately evaluate power consumption, we employ a MakerHawk USB-type digital multimeter [50], as illustrated in Figure 8. This multimeter offers approximately 1% accuracy for both current and voltage measurements and covers a range of 0–5 A and 4–30 V. Our experimental hardware platform, the ESP32-S3 microcontroller, is powered via the serial port of a PC, receiving an external input voltage of 5 V, which is internally regulated down to 3.3 V to power the MCU core. Current consumption values reported in official datasheets and benchmark studies typically reference this internal operating voltage (3.3 V), as it directly powers the microcontroller [51]. Therefore, to precisely estimate the ESP32-S3’s MCU-specific power consumption during inference and Wi-Fi communication, we measure the current draw using the multimeter and subsequently calculate power consumption by multiplying the measured current by the MCU’s internal voltage of 3.3 V.

We first measure power consumption for each node within the distributed pipeline. Subsequently, we evaluate the power consumption of the quantized LeNet model and the baseline original model. For the baseline model, we require a device with sufficient internal memory, as using external PSRAM may introduce additional variables and inaccuracies in power measurement. The authors in [52] have previously demonstrated the successful deployment of an uncompressed LeNet-5 CNN (approximately 245 KB in size) entirely within the 1 MB internal SRAM of an STM32H7 MCU without relying on external memory. Given the similarity in model size to our 226 KB baseline model, the STM32H7 MCU serves as an appropriate platform for accurately measuring the baseline model’s power consumption. Thus, we select the STM32H7 as the reference device for evaluating and comparing the power efficiency of our DDSNN approach against a conventional, non-distributed deployment.

### 5.5. Distributed Multi-Source Anomaly Detection

As introduced earlier in Section 3.3, we have two distinct pipelines: our main inference pipeline and the raw data stream pipeline designed for transmitting raw data from downstream nodes back to the head node (Node 1). For the main pipeline, our primary evaluation focuses solely on ensuring that the data collected on Node 1 passes smoothly through the pipeline. Since the pipeline structure remains unchanged and is not specifically modified for this additional configuration, we do not directly assess accuracy. Instead, our primary objective is to confirm that all collected data successfully traverses the pipeline, enabling predictions for each sample. Ensuring smooth data traversal inherently maintains accuracy and inference performance consistent with the primary pipeline setup. To accomplish this, we divided our test dataset, comprising a total of 606 samples, into three equal segments, each consisting of 202 samples. These segments were integrated into Nodes 1, 2, and 3 deployment scripts, respectively. Upon arrival at Node 1, each sample is assigned a unique identifier based on its originating node: samples from Node 1 are numbered from 1 to 201, samples from Node 2 are numbered from 202 to 403, and samples from Node 3 receive IDs ranging from 404 to 606. This identification strategy allows us to precisely determine how many samples successfully completed their journey through the entire pipeline at the final node.

Regarding the parallel raw data stream pipeline, we measure latency for each node starting from the moment data is transmitted by the originating node, through each pipeline stage, until a prediction is generated at the final node. To prevent any interference with the main pipeline, distinct ports are assigned to each node for transmitting raw data from downstream nodes (Nodes 2 and 3). This configuration operates concurrently with the main inference pipeline, effectively creating an additional feedback loop. Nodes 2 and 3 independently transmit raw test data back to Node 1, which then integrates these additional data streams into the primary pipeline. To mitigate the risk of Node 1 becoming overwhelmed by continuous incoming data from multiple sources, we implemented deliberate timing delays in the raw data transmissions from Nodes 2 and 3. These controlled intervals effectively prevent overload and potential pipeline stalls, ensuring stable and continuous operation. The latency for each node is specifically defined as follows:

L=Main inference latency+Raw data sending latency+Controlled delays

- Main inference latency is the duration required to process data within the main inference pipeline.
- Raw data sending latency is the transmission time from the originating node to the head node.
- Controlled delays refer to intentionally introduced timing intervals at raw data sending-nodes to regulate data flow and prevent the head node from being overloaded.

## 6. Results and Discussion

**Figure 9:**
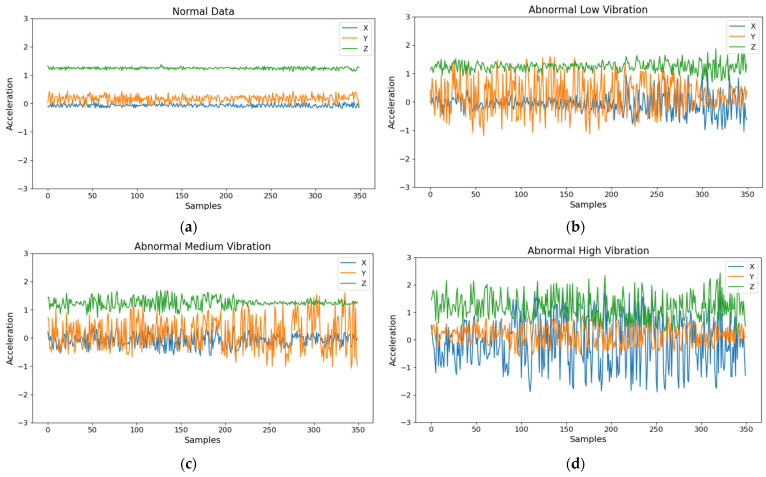
Example accelerometer data samples: (**a**) normal vibration; (**b**) abnormal vibration (low intensity); (**c**) abnormal vibration (medium intensity); (**d**) abnormal vibration (high intensity).

This section presents the results obtained from evaluating the performance of our DDSNN, quantized int8 LeNet model, and original LeNet model as a baseline. We report metrics such as inference accuracy under binary and multi-class classification scenarios; pipeline throughput; and latency for DDSNN, int8 quantized LeNet, and original LeNet. We collected a total of 125,600 normal samples and 177,000 abnormal samples, which we visualized using Matplotlib according to their respective categories. Figure 9 illustrates examples of normal data samples alongside abnormal data samples at three distinct levels of vibration intensity: low, medium, and high.

**Figure 10:**
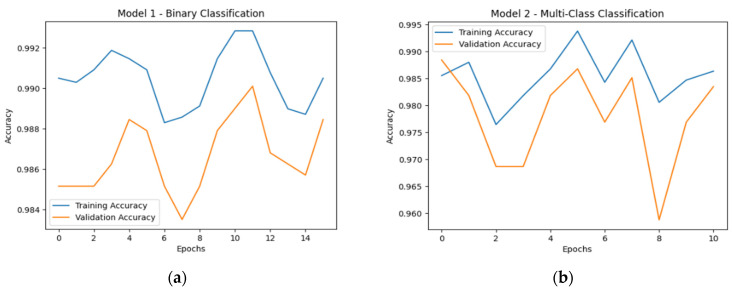
Baseline LeNet model training and validation accuracy over epochs: (**a**) binary classification model; (**b**) multi-class classification model.

The baseline LeNet model achieves an accuracy of 99.01% in the binary classification task and 98.84% in the multi-class classification task. Figure 10 illustrates each scenario’s corresponding training and validation accuracy across epochs.

### 6.1. Inference Accuracy

**Figure 11:**
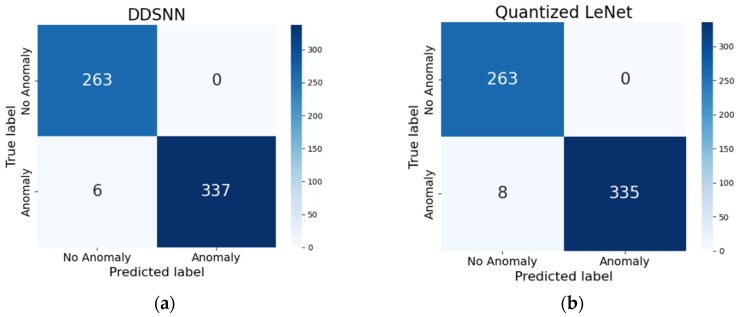
Confusion matrices for binary classification: (**a**) baseline and DDSNN models; (**b**) quantized LeNet model.

We first evaluated the models under the binary classification task. The binary test set consisted of 606 samples (343 abnormal, 263 normal). Because our DDSNN exactly replicated the baseline model’s predictions, the confusion matrix shown in Figure 11a represents both DDSNN and the baseline model. Consequently, the DDSNN precisely maintained the baseline accuracy of 99.01%. In contrast, the quantized LeNet model exhibited a slight reduction in accuracy, achieving 98.68%, as detailed in Table 2 and illustrated in Figure 11b.

**Figure 12:**
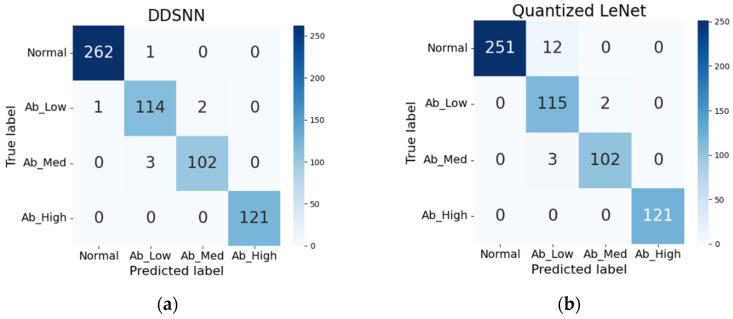
Confusion matrices for multi-class classification: (**a**) baseline and DDSNN models; (**b**) quantized LeNet model.

In the multi-class scenario, our DDSNN approach once again precisely replicates the performance of the baseline LeNet model, achieving an accuracy of 98.84% with identical predictions, as shown in Figure 12a and detailed in Table 2. However, the quantized LeNet model, which performed closely to the baseline in the binary classification, showed a noticeable drop in accuracy for the more complex multi-class task, achieving 97.19% with a 1.65% accuracy loss. The confusion matrix in Figure 12b indicates that this model misclassified 11 low-vibration anomaly samples as normal, reducing its precision to 96.64%. These results suggest that 32-bit precision is crucial for maintaining accuracy in more complex classification tasks.

Table 2 summarizes the performance metrics for DDSNN and int8-LeNet. These results clearly demonstrate that DDSNN reliably maintains accuracy across both classification tasks during on-device inference.

### 6.2. Inference Latency

According to our fixed delay-based flow management defined in 3.4, we first determine the fixed delay by identifying the bottleneck node within our distributed inference pipeline based on the computational time and transfer time of each node. To do this, we calculate each node’s computational time and data transfer time based on intermediate activation sizes. Specifically, we derive intermediate activation sizes from the output shapes of our partitions shown in Figure 6.

- Node 1 to Node 2: 48×64 floats≈3072 floats×4 bytes≈12 KB
- Node 2 to Node 3: 736 floats ×4 bytes ≈2944 bytes ≈2.9 KB

Transfer time $L_{i}$ is determined as follows:

(12) $$L_{i}\left(ms\right)=\frac{Activation\;Size\;(KB)}{Bandwith\;(KB/s)}\times 1000$$

Our Wi-Fi network operates on the 802.11n standard at 2.4 GHz with a 40 MHz channel bandwidth. This configuration offers a maximum theoretical PHY data rate of around 150 Mbps. However, practical throughput is typically lower due to overhead, interference, and real-world conditions. Therefore, we assume a practical throughput of about 50–60% of this theoretical rate, resulting in approximately 75 Mbps (roughly 9375 KB/s). Based on these parameters, we calculated the transfer time and total service time for each node, along with the practically measured computational time, as shown in Table 3.

As can be noticed from the table, Node 2 service time becomes a bottleneck $T_{max}=80.31\;ms$ and pipeline depth in our case W=3, thus:

$$SEND\text{\_}DELAY=\frac{80.31}{3}\approx 27\;ms$$

Although this is a theoretical assumption, a realistic value must be fine-tuned experimentally to achieve optimal performance. If the delay is too low, input data is transmitted faster than downstream nodes can process, resulting in overwhelmed buffers and excessive computational overhead. This situation leads to pipeline freezes, causing interruptions in data flow and ultimately resulting in lost samples that compromise overall inference accuracy. On the other hand, setting the delay too high introduces unnecessary waiting periods between transmissions, thereby increasing overall latency without benefiting performance. Through careful experimentation, we optimized the delay to 30 ms, achieving reliable performance where all 606 test samples passed smoothly through the pipeline without loss. We also evaluated DDSNN’s inference throughput by allowing a pure TCP flow control mechanism without employing any controlled delay methods, following the approach implemented in previous studies [38,40]. Figure 12 shows a comparison of our controlled approach using the optimized fixed-delay method versus the TCP built-in flow control method.

**Figure 13:**
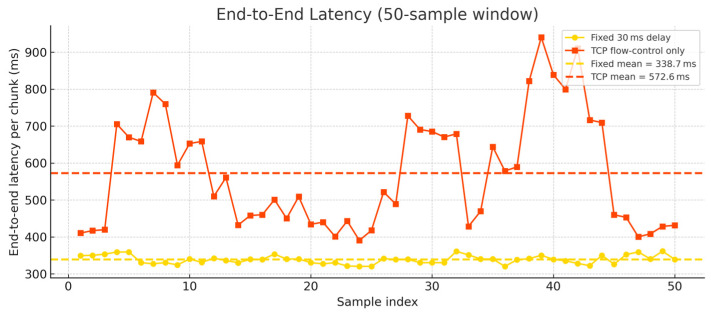
Comparison of end-to-end latency for DDSNN under two flow-control strategies—optimized application-layer fixed delay and pure TCP flow control. The graph shows the latency for 50 practical inference samples per method, highlighting that the fixed-delay method achieves significantly lower average latency (338.7 ms) with less variability compared to TCP’s built-in flow control mechanism (572.6 ms).

As illustrated in Figure 13, our fixed delay-based method demonstrates lower end-to-end latency per sample compared to the TCP flow control method. According to the latency calculations summarized in Table 4, the theoretical latency for each sample, represented by the sum of total service times, initially suggested that the TCP-based flow control would outperform our fixed-delay approach. This is because our method intentionally introduces small intervals between data chunks, thereby adding deliberate delays to the overall latency to prevent downstream nodes from becoming overwhelmed. However, practical experimental results revealed that our optimized fixed-delay method significantly outperformed the TCP flow control method. Specifically, our method achieved an average inference latency of 338.7 ms, whereas the TCP flow control method yielded a considerably higher latency of 572.6 ms with noticeable and consistent fluctuations.

This fluctuation results from TCP’s inherently reactive design, where congestion and varying network conditions trigger frequent adjustments of transmission windows and acknowledgments. Such dynamic, packet-level flow adjustments create substantial overhead and unpredictability, especially on resource-constrained microcontrollers. Consequently, TCP’s built-in flow control method results in higher inference throughput with 133.13 ms per chunk. Whereas our fixed delay approach achieves a notably lower inference throughput of 102.45 ms, as shown in Table 4. Additionally, the pipeline utilizing the TCP flow-control method completed processing 606 samples in 80.145 s (7.56 samples per second), whereas employing our fixed-delay method substantially reduced the total completion time to 61.676 s (9.82 samples per second).

**Figure 14:**
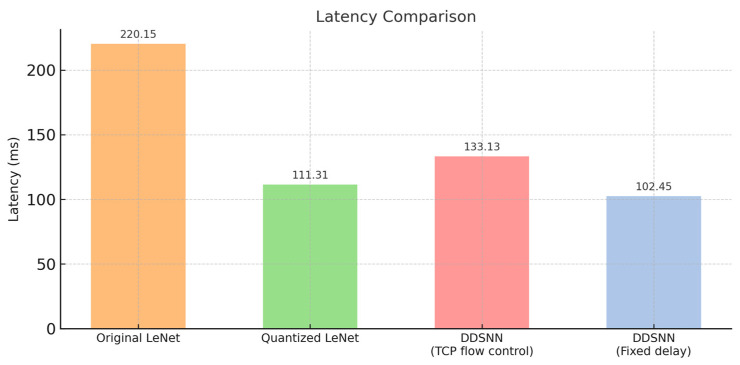
Latency comparison of original LeNet, quantized LeNet, and DDSNN with TCP flow control method and with the proposed fixed-delay method.

For further comparison, we use the inference throughput shown in Figure 13, as it is the latency achieved through pipeline parallelism, where each node processes input data simultaneously to accelerate inference throughput. We then experimentally measured non-partitioned LeNet and quantized int8 LeNet latency and compared them against the inference throughput achieved. Figure 14 compares latency performance across four scenarios: the original non-partitioned LeNet (baseline), a quantized LeNet, and our proposed DDSNN implemented with two pipeline dataflow management strategies—our proposed fixed-delay controlled method and TCP’s built-in flow control approach. The original LeNet exhibits the highest latency (220.15 ms per inference), highlighting the inefficiency of the non-partitioned model in latency-sensitive scenarios. Quantized LeNet significantly improves latency (111.31 ms), demonstrating the impact of quantization methods in reducing inference time. The DDSNN using TCP flow control achieves 133.13 ms, outperforming the original LeNet but still falling behind the quantized model. Importantly, our DDSNN employing the proposed fixed-delay approach achieves the lowest latency (102.45 ms), outperforming both quantized LeNet and the TCP-controlled DDSNN, delivering more than twice the latency reduction compared to the original LeNet model.

Ultimately, our practical findings underline the importance of considering realistic hardware constraints and network dynamics rather than relying solely on theoretical throughput calculations, especially when deploying distributed inference pipelines on tiny microcontrollers. While our proposed method outperforms the other scenarios, the intermediate “forwarding” node in our partitioned LeNet could potentially become a performance bottleneck. This possibility arises from the node’s dual responsibilities: receiving intermediate activations from the preceding node and immediately processing and forwarding its outputs downstream. As a result, its computation time is inherently longer compared to other nodes. If this processing time significantly exceeds that of the preceding node, it could introduce additional latency and slow down the overall progression of data through the pipeline, thereby limiting overall throughput. In principle, assigning more computation to Node 1 rather than to the intermediate node would be preferable since a front-loaded workload prevents downstream devices from being overwhelmed. However, our design also supports a Distributed Multi-Source Anomaly Detection configuration in which Node 1 must handle raw data from several peer sensors before feeding them to the distributed pipeline. To keep that front-end task responsive, we retain the current layer split even though it leaves the intermediate node with the heaviest load. Although alternative partition points could be examined to reduce the workload on this node, potentially lowering inference latency even further, our primary goal is to maintain high accuracy at the edge rather than maximizing inference throughput. Overall, the current DDSNN latency is acceptable and still the best among the setups shown in Figure 14.

### 6.3. Power Consumption

**Figure 15:**
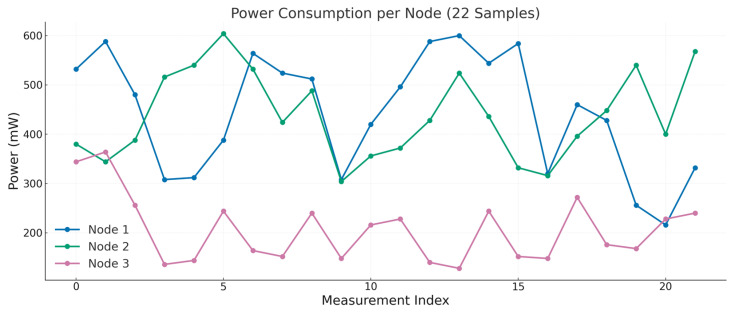
Power consumption per node for the distributed pipeline inference on ESP32-S3 Microcontrollers.

For the distributed pipeline, we measured the power consumption of 22 inference samples per node, as illustrated in Figure 15. The per-node power consumption encompasses all inference-related operations, including tensor computations and wireless communication tasks such as sending and receiving intermediate results. It is important to note that the power measurements discussed here only reflect the energy consumed during the primary inference process and do not include power usage related to transmitting raw data, as this is considered an additional setup specifically for predictive maintenance beyond our main DDSNN approach. The results indicate noticeable differences in energy usage patterns across nodes, reflecting the impact of neural network structure complexity and wireless communication loads on power demand.

Node 1, which executes the initial and largest convolutional layers and transmits intermediate feature maps over Wi-Fi, exhibited the highest power consumption, averaging approximately 377 mW and peaking around 508 mW. Node 2, despite featuring a reduced convolutional structure compared to Node 1, showed a similar energy pattern, averaging about 361 mW with peaks near 498 mW. This suggests that Node 2’s energy usage is significantly influenced by its additional communication responsibilities for receiving data from Node 1 and transmitting processed data onward to Node 3. Conversely, Node 3, primarily handling dense layers with substantially less computational and communication demands, demonstrated notably lower power consumption, averaging approximately 170 mW with peaks of around 300 mW. This observation aligns with prior studies showing that convolutional layers account for roughly 72% of a CNN’s inference energy, whereas the later fully connected layers contribute only about 28% [53]. Notably, the brief surge to ~0.15 A (≈500 mW at 3.3 V) on Nodes 1 and 2 is consistent with known Wi-Fi transmission spikes on the ESP32-S3 platform, indicating that intermediate result forwarding is a major factor in those power peaks. Such wireless communication tasks, especially Wi-Fi data transfers, are widely recognized as substantial contributors to overall power usage in wireless sensor networks, often rivaling or surpassing computational energy consumption.

Nevertheless, even at their maximum, the per-node power levels remain within the practical range of typical battery-powered IoT devices (sub-watt scale), reinforcing the feasibility of deploying the distributed inference pipeline in real-world WSN environments. Existing literature confirms that wireless sensor nodes typically operate at power levels ranging from a few milliwatts up to a few hundred milliwatts [54]. Specifically, prior research [55] reports that the ESP32-S3 consumes 45 mA when not using Wi-Fi and 120 mA while transmitting data over Wi-Fi, and highlighted that transmitting data (2.4 GHz 802.11n) causes a large spike in current draw. According to ESP32-S3’s datasheet [56], during active Wi-Fi transmission, the device can spike to peak currents around 283–340 mA (at 3.3 V) for sending data, depending on the 802.11 mode and transmit power.

In contrast, the quantized LeNet model showed significantly lower power consumption, ranging from 72.6 to 102.3 mW, confirming the substantial power efficiency gains achievable with model quantization. Prior research emphasized that the ESP32-S3’s advanced hardware features, such as optimized cache and SIMD vector instructions, contributed to energy reductions of up to 85% per inference, particularly when employing quantized models [51].

To benchmark the original baseline LeNet model, we selected the STM32H743 due to its sufficient internal memory capacity. According to the STM32H743 datasheet provided by STMicroelectronics (2023) [57], operating at its maximum frequency of 480 MHz, the microcontroller typically consumes around 110 mA at 3.3 V, translating to a power consumption of approximately 363 mW, with a maximum current of 129 mA (425.7 mW). However, empirical studies have reported slightly higher power consumption during active inference, typically ranging between approximately 500–615 mW, dependent on the complexity of the executed ML model. Specifically, the authors in [52] benchmarked several popular ML models on the STM32H743 microcontroller, reporting that running the uncompressed LeNet-5 model (approximately 245 KB) required approximately 606.6 mW, while the quantized version (72 KB) significantly reduced the consumption to 545.6 mW. Furthermore, the Simple Sine Calculation Model (3 KB) demonstrated lower power consumption, around 526.2 mW in its uncompressed form and approximately 524.6 mW when employing quantization-aware training.

**Figure 16:**
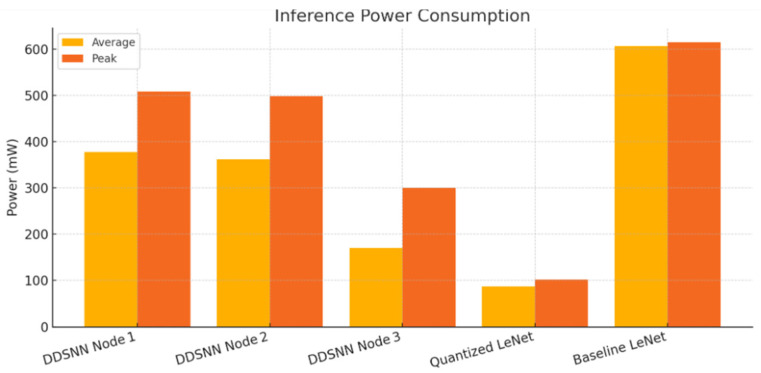
Average and peak inference power consumption for DDSNN nodes (ESP32-S3 MCUs) compared to baseline and quantized LeNet models. Baseline LeNet measurements on STM32H7 are drawn from [52].

Finally, the measured energy consumption for each DDSNN node, along with the quantized LeNet and baseline LeNet power estimates, is presented in Figure 16. Notably, 8-bit quantized LeNet running on the same hardware consumed significantly less power, only on the order of 70–100 mW during inference, thanks to smaller arithmetic operations and efficient use of the ESP32-S3’s optimized SIMD instructions. However, this improvement in energy efficiency comes with reduced model accuracy. In our experiments, maintaining full 32-bit precision allowed significantly higher accuracy, especially for complex multi-class classification, compared to the quantized model. Thus, from an application perspective, there is a clear accuracy-versus-energy trade-off. For scenarios where ultra-low power operation is paramount and a slight accuracy loss is acceptable (e.g., long-term monitoring on a tight energy budget), a quantized single-model deployment may be preferable. On the other hand, when high accuracy and fast responses are critical, our results indicate that distributed full-precision inference using DDSNN on microcontrollers is a practical choice. While it does consume more power than the quantized approach, it still uses significantly less energy compared to the original baseline model.

In summary, the power consumption results demonstrate that our full-precision DDSNN can run on low-power microcontrollers without exceeding their power budgets, but they also reveal opportunities for optimization, for example, balancing the workload or refining communication strategies to mitigate the heavy energy demand observed in the early stages of the pipeline and optimizing energy use using WSN-friendly communication methods.

### 6.4. Latency and Accuracy Comparison

**Figure 17:**
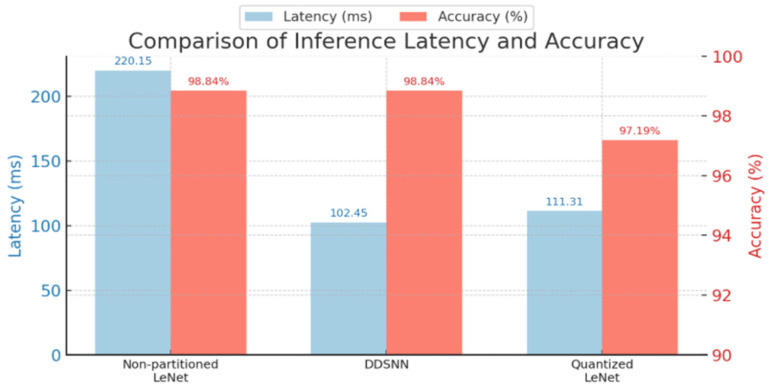
Comparison of latency and accuracy across three deployment methods: non-partitioned original LeNet, DDSNN, and quantized int8 LeNet.

In this section, we summarize the performance metrics from our experiment and compare inference latency along with accuracy for three models: baseline LeNet, our DDSNN, and a quantized int8 LeNet model, as shown in Figure 17. The DDSNN demonstrates the best latency performance, achieving 102.45 ms per inference, over two times faster than the original non-partitioned LeNet at 220.15 ms, while maintaining identical accuracy (98.84%). Although the quantized LeNet achieves competitive latency (111.31 ms), its accuracy slightly drops to 97.19%, indicating a trade-off between speed and precision.

### 6.5. Multi-Source Anomaly Detection

**Figure 18:**
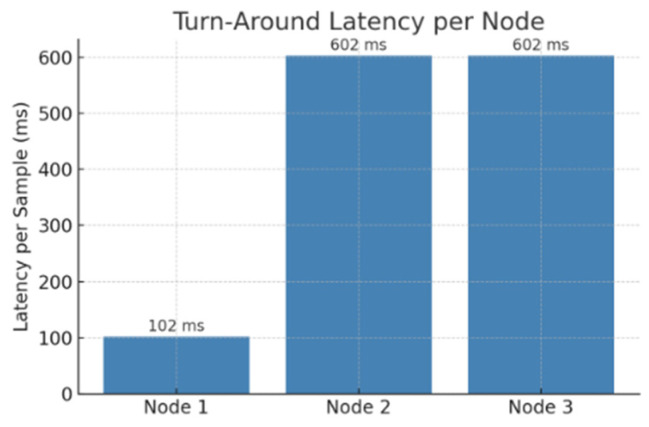
Turnaround latency per node represents the total time required for vibration data collected at each node to pass through the distributed pipeline, from initial data collection to final anomaly prediction, in the multi-source anomaly detection setup.

We first introduced intentional transmission delays at downstream nodes to prevent the head node (Node 1) from becoming overwhelmed. Through experimental optimization, we found that a delay of 250–300 ms ensured smooth data flow without causing pipeline freezes. When the delay was set too short, it resulted in some data loss across the pipeline, preventing samples from reaching the final node. This optimization achieved excellent results, with all 202 samples from each node successfully traversing the pipeline and reaching the final node without any loss. Next, we measured the raw data sending time from downstream nodes back to the head node, finding it approximately 200 ms per sample. We then calculated the total latency per node based on these experimentally determined measurements. For Node 1, there was no additional raw data sending latency since it directly feeds its raw data into the pipeline, resulting in a total latency of approximately 102 ms. For Nodes 2 and 3, as defined in Section 5.5, the total latency per sample is the sum of the main pipeline inference time (102 ms), the measured raw data transmission latency (200 ms), and the deliberately introduced delay (300 ms), resulting in a total latency of approximately 602 ms for each node. Figure 18 illustrates the turnaround latency per node when implementing this additional multi-source detection setup.

As can be observed, Node 1 with direct data integration achieves significantly lower latency compared to Nodes 2 and 3, which incorporate additional delays and transmission times. We believe that the observed latency is fully acceptable for applications without strict real-time requirements. Specifically, in predictive maintenance scenarios, anomalies typically develop gradually over seconds to minutes [58]. This gradual progression provides sufficient time for early detection and timely response, enabled by our distributed anomaly detection setup. This approach demonstrates how other nodes can be effectively utilized in parallel with the main inference pipeline. The delays introduced at downstream nodes can be further optimized depending on specific priorities: shorter delays can be implemented if reduced latency is preferred, potentially at the cost of minor data loss.

### 6.6. Limitation

While our DDSNN approach offers several advantages for deploying deep neural networks on resource-constrained microcontrollers, some limitations exist that should be considered. First, although the decentralized architecture eliminates the need for a central dispatcher, it remains inherently susceptible to individual node failures. Specifically, if any node in the pipeline goes offline, the inference process temporarily halts due to the sequential nature of our system. Currently, DDSNN does not include an automatic recovery mechanism or dynamic reassignment of model partitions during runtime. Instead, partitions are statically deployed to nodes prior to operation, which restricts automatic adaptability in the event of node failures or changes in network conditions.

Nevertheless, this static deployment has practical advantages: recovery from node failure can be quickly achieved by simply replacing the malfunctioning node without extensive reconfiguration. In contrast to other synchronization-dependent methods (e.g., [38,40]), which require complex adjustments across nodes due to tight inter-layer dependencies, DDSNN enables straightforward replacement of individual nodes with minimal disruption to the overall system. Future enhancements could explore dynamic deployment strategies or adaptive partitioning methods that would allow DDSNN to automatically handle node failures or adapt to changing runtime conditions, thus improving robustness and flexibility.

Another limitation could be the dependence of our method on wireless communication for forwarding intermediate results between nodes. In environments with challenging or unreliable communication conditions, such as industrial settings or remote outdoor deployments, performance may degrade, resulting in potential accuracy loss or interruptions in inference due to dropped transmissions or interference. Therefore, future research should specifically target the evaluation and optimization of DDSNN performance under adverse communication conditions, ensuring its robustness and reliability even in challenging deployment environments.

## 7. Conclusions and Future Work

In this study, we introduced DDSNN, a novel fully decentralized DNN inference method tailored for TinyML in WSNs. By partitioning a pre-trained LeNet across three low-power ESP32-S3 microcontrollers without compression, DDSNN overcomes the memory and computation constraints that traditionally hinder deploying full-precision DNNs on tiny devices. Our end-to-end design incorporates a lightweight flow-control mechanism at the application layer to manage the pipeline of intermediate data, preventing bottlenecks and ensuring smooth sequential processing across nodes. We deployed this system on an actual WSN testbed of three ESP32-S3 microcontrollers in a distributed anomaly detection scenario (industrial pump vibration monitoring), demonstrating its practical feasibility in a real-world setting. The experimental results confirm that DDSNN maintains the full accuracy of the original model during on-device inference, exactly matching the non-distributed baseline’s accuracy (≈99% on binary classification; 98.84% on multi-class). Notably, preserving full 32-bit precision allowed DDSNN to outperform a quantized int8 model in complex tasks. For example, in a multi-class anomaly detection scenario, the quantized model’s accuracy dropped to 97.19%, whereas DDSNN retained the baseline’s 98.84%. At the same time, DDSNN achieved superior inference speed: end-to-end latency was ~102 ms per sample, over 2× faster than the Baseline LeNet running on a single MCU (~220 ms). This latency also slightly outpaced the quantized model (~111 ms), indicating that our sequential multi-MCU pipeline not only preserves accuracy but also delivers real-time performance.

In terms of energy performance, distributing the workload across nodes yielded a reduction in per-node energy consumption compared to the original single-device approach. Although a heavily quantized model consumed the least power overall, it did so at the cost of accuracy. In contrast, DDSNN provides a compelling balance by retaining full-precision accuracy with reasonable energy consumption, achieved by distributing computation-intensive layers across separate nodes and reducing idle time between processing stages. These results highlight that sequentially distributed inference can provide the best of both worlds: the low latency typically seen in optimized or parallel models (pipeline parallelism), together with the high accuracy of a full-scale network, all on highly resource-constrained hardware. This successful deployment of DDSNN in a realistic WSN underscores its capacity to expand the horizons of TinyML, enabling more complex and high-precision edge intelligence in scenarios where traditional single-node or cloud-based solutions would be impractical.

Looking forward, several avenues can further enhance DDSNN’s performance and robustness. First, dynamic partitioning and scheduling strategies should be investigated to adaptively distribute the neural network layers based on node capabilities or runtime conditions, thereby balancing the load (especially on intermediate nodes) and optimizing overall throughput. Second, Power consumption analysis indicates that the computational load and communication overhead in DDSNN are primarily concentrated in the convolution-intensive early nodes (Node 1 and Node 2). This highlights opportunities for further optimization, such as redistributing computational tasks more evenly across nodes or adopting energy-efficient, WSN-friendly communication protocols like Bluetooth Low Energy or Zigbee.

Third, rigorous robustness testing under adverse network conditions (e.g., interference, high latency links, or even node failures) is needed to ensure DDSNN maintains reliable inference in industrial and remote deployments. Such experiments will guide optimizations (e.g., more resilient flow control or error-handling mechanisms) to strengthen DDSNN’s fault tolerance. Additionally, Additionally, our efficient memory utilization, achieved by distributing computational loads across multiple nodes, allows future exploration of on-device training methods such as tabular or tiny-DQN Q-learning. These sequential learning methods align naturally with DDSNN’s pipeline structure, enabling local learning and enhancing adaptability to changing environments, thereby further strengthening overall system robustness. By addressing these areas—adaptive partitioning, energy-efficient networking, thorough stress-testing, and potential on-device learning extensions—we aim to further solidify DDSNN as a foundation for next-generation TinyML solutions in wireless sensor networks, combining accuracy, efficiency, and resilience in real-world deployments.

## Figures and Tables

**Table 2:** Performance metrics comparison of DDSNN and quantized (int8) LeNet models against the baseline for binary and multi-class classification tasks.

Class Type	Metrics				
	Model	Accuracy (%)	Precision (%)	Recall (%)	F-Score (%)
Binary	Baseline	99.01	100	98.25	99.1
	DDSNN	99.01	100	98.25	99.1
	Quantized LeNet	98.68	100	97.66	98.8
Multi-Class	Baseline	98.84	98.58	98.55	98.65
	DDSNN	98.84	98.58	98.55	98.65
	Quantized LeNet	97.19	96.64	97.72	97.08

**Table 3:** Total node service times.

Node	Computation $\left(C_{i}\right)$	Transfer $\left(L_{i}\right)$	Total Service $\left(T_{i}\right)$
Node 1	26 ms	1.28 ms	27.28 ms
Node 2	80 ms	0.31 ms	80.31 ms
Node 3	2.65 ms	-	2.65 ms

**Table 4:** Comparison of two scenarios for DDSNN when using TCP’s built-in flow control and fixed-delay flow management for pipeline parallelism.

Metrics	TCP Flow Control	Fixed Delay	Improvement
Inference throughput per sample (ms)	133.13	102.45	↓ 23.1%
Total time for 606 samples (s)	80.145	61.676	↓ 23.1%
Samples per second	6.56	9.82	↑ 30.0%

Percentage change is relative to the TCP baseline (lower is better for latency/total time, while higher is better for throughput).

## Data Availability

The dataset generated and analyzed during the current study is publicly available at [PredictiveMaintenance_Vibration_WaterPump_2025-v1.0]. The data comprises accelerometer measurements collected from an industrial motor operating under various vibration intensities, categorized into five operational scenarios: Motor Off, Normal Operation, Abnormal Low, Abnormal Medium, and Abnormal High. Each data sample includes timestamped vibration readings along the X, Y, and Z axes at a sampling rate of 100 Hz. The dataset can be accessed via the following link: [https://github.com/Airnazar/PredictiveMaintenance_Vibration_WaterPump_2025-v1.0-] (accessed on 21 May 2025).

## Abbreviations

The following abbreviations are used in this manuscript:

DDSNN	Decentralized Distributed Sequential Neural Network
TinyML	Tiny Machine Learning
DNN	Deep Neural Network
MCUs	Microcontroller Units
WSNs	Wireless Sensor Networks
TCP	Transmission Control Protocol
ACK	Acknowledgement
EC	Edge Computing
CNN	Convolutional Neural Network
LSTM	Long Short-Term Memory
SRAM	Static Random Access Memory
PSRAM	Pseudo Static Random Access Memory
WMN	Wireless Mesh Network
